# Transgender Veterans and the Veterans Health Administration: Exploring the Experiences of Transgender Veterans in the Veterans Affairs Healthcare System

**DOI:** 10.1089/trgh.2016.0006

**Published:** 2016-06-01

**Authors:** Kris Rosentel, Brandon J. Hill, Connie Lu, Joshua Trey Barnett

**Affiliations:** ^1^ Department of Obstetrics and Gynecology, Center for Interdisciplinary Inquiry and Innovation in Sexual and Reproductive Health (Ci3), The University of Chicago, Chicago, Illinois.; ^2^ School of Social Service Administration (SSA), The University of Chicago, Chicago, Illinois.; ^3^ Department of Gender and Sexuality Studies, The University of Chicago, University of Chicago, Illinois.; ^4^ Section of Family Planning and Contraceptive Research, Department of Obstetrics and Gynecology, The University of Chicago, Chicago, Illinois.; ^5^ The Kinsey Institute for Research in Sex, Gender, and Reproduction, Indiana University, Bloomington, Indiana.; ^6^ Department of Psychological and Brain Sciences, Indiana University, Bloomington, Indiana.; ^7^ Department of Gender Studies, Indiana University, Bloomington, Indiana.; ^8^ Department of Communication, University of Utah, Salt Lake City, Utah.

**Keywords:** military/Department of Defense, transgender, transgender health, transgender veterans, VA clinic

## Abstract

**Purpose:** The Veterans Health Administration (VHA) has provided transgender transition-related care to veterans since 2011. However, little is known about the experiences of transgender veteran patients accessing transgender transition-related healthcare at Veterans Affairs (VA) clinics since the establishment of this care. The purpose of this study was to explore transgender veterans' experiences accessing and utilizing transition-related healthcare through the VA healthcare system.

**Methods:** Eleven transgender veterans were recruited using in-person recruitment at the 2013 Southern Comfort Conference (Atlanta, GA). In-depth semistructured interviews were conducted with participants using a qualitative inquiry methodological perspective and experience-centered approach. Interviews were digitally recorded and transcribed verbatim. The transcripts were coded by two independent researchers using ATLAS.ti.©

**Results:** Five inter-related themes were identified as key factors impacting the accessibility and quality of care transgender veterans receive through the VA: (1) long delays in receiving care; (2) needing to travel to receive care; (3) lack of patient knowledge regarding the coverage of transition-related care; (4) insensitivity, harassment, and violence among providers; and (5) a general lack of knowledge about transgender patients and care among providers.

**Conclusion:** To our knowledge, this study is one of the first to explore the experiences of transgender veterans in accessing and utilizing transgender transition-related care at the VA after the 2011 VHA directive. Our findings suggest that although transgender healthcare coverage is available, additional patient-centered and healthcare system-level interventions are needed to improve the uptake and implementation of the VHA transgender protections and care coverage.

## Introduction

Although transgender people are currently barred from openly serving in the United States (US) military, the Veterans Affairs (VA) healthcare system has been providing transition-related and gender-affirming care to transgender veterans since 2011. On June 9, 2011, the Department of Veterans Affairs, Veterans Health Administration (VHA) issued a directive, “Providing Healthcare for Transgender and Intersex Veterans,” which established the provision of hormone therapy, gender-related mental health counseling, and other transition-related services through the VA, as well as a mandate that the VA health system provides care “without discrimination and in a manner … consistent with the Veteran's self-identified gender.”^1,2^ This directive, however, does not include coverage of surgical procedures although the VA does provide transgender veterans with pre- and postoperative care.^1,2^

The VHA directive constitutes a major step toward improving the health of transgender veterans, who face significant health disparities.^3–6^ Although research suggests that the transgender population in general experiences severe physical and mental health disparities, compared to the cisgender population, including high rates of HIV, suicidality, depression, anxiety, and mental health-related hospitalization,^7–9^ these disparities are even more glaring among transgender veterans.^3–6^ In a recent survey of transgender veterans and transgender active-duty service members, transgender veterans reported several mental health diagnoses, including depression (65%), anxiety (41%), PTSD (31%), and substance abuse (16%).^10^ In a study by Blosnich et al. examining VHA patient records from 2000 to 2011 (before the 2011 VHA directive), the rate of suicide-related events among veterans with a gender identity disorder (GID) diagnoses was found to be 20 times higher than that of the general VHA patient population.^3^ However, it is important to note that not all transgender-identified veterans necessarily meet criteria for GID or gender dysphoria.^10^ In another study assessing suicide mortality among transgender veterans, Blosnich et al. found that the crude rate of suicide among veterans with transgender-related ICD-9-CM diagnoses (82/100,000 person-year) was higher than the crude rates for both the VHA and U.S. population in general.^4^ Taken together, these findings suggest high rates of mental health conditions among transgender veterans, compared to transgender active-duty service members, non-transgender veterans, and the general non-transgender population.

In addition, recent research has demonstrated that transgender people face several barriers to accessing healthcare, including discrimination, limited coverage, lack of knowledge among providers, and experiences of harassment and violence in medical settings.^5,7,8^ In a large national survey of 827 transgender veterans, conducted by the Transgender American Veterans Association (TAVA) and Palm Center, researchers found that more than one in five transgender veterans who sought or received healthcare through the VA reported discriminatory treatment by doctors and staff before the 2011 VHA directive.^6^ Through establishing protections against discrimination and expanding the level of care, the VHA has taken several steps to address some of the most persistent challenges transgender veterans face in healthcare access. More specifically, the establishment of transition-related care provision, such as hormone therapy, may be particularly significant for transgender veteran health as this care has been found to alleviate distress associated with gender identity and bodily incongruence for many transgender people.^11–14^

Given that transgender people are over-represented in the US veteran community, the VHA recent establishment of care to transgender veterans is significant for both the fields of transgender health and veteran health in general.^15–19^ Population-level data from the National Transgender Discrimination Survey estimate that roughly 134,300 transgender individuals, who comprise around 19% of the transgender population in the United States, hold veteran or retired National Guard or Reserve status from the US military.^5,20,21^ Research also indicates that a significant portion of transgender veterans utilize the VA for care, which suggests that the VA healthcare system is one of the largest healthcare providers for transgender people in the United States.^22^ A study by Shipherd et al. found that about 30% of female-identified transgender veterans surveyed in 2008 had sought medical or mental healthcare through the VA during the previous 6 months.^18^ Two additional studies suggest that the number of transgender veterans seeking care through the VA healthcare system is increasing.^3,23^

Despite the VHA efforts to expand coverage for transgender patients over the past 5 years, little research has specifically examined the VA implementation of transgender veteran healthcare services using a patient-centered approach. Findings from a recent study of LGBT patients at two southern VA hospitals suggested that provider–patient communication may be a persistent issue as most respondents had never been asked about their sexual orientation (62%) or gender identity (81%).^24^ In addition, research examining sexual and gender minority (SGM) veterans has also recommended that better documentation of the prevalence of SGM veterans, an increased understanding of the unique context and needs of SGM veterans, and improved cultural competency among VHA providers regarding SGM veterans are needed to improve the VA healthcare system for SGM veterans.^22^ Furthermore, Johnson et al. have argued that improving mental health provider competency may be critical as VA mental health professionals must be equipped to meet the overlapping mental health needs of transgender veterans in relation to gender dysphoria, minority stress, and identity concealment.^25^

To this end, the research by Lutwak et al. has suggested that many providers, VA and non-VA alike, still lack the adequate training to address the specific healthcare needs of transgender veterans, signifying the need for additional training and interventions.^26^ Surveys conducted by Sherman et al. shortly after the directive were further initiated to support the need for specialized training as they found that the majority of surveyed VA providers at two VA hospitals—Houston, Texas, and Oklahoma City, Oklahoma—had not received any training or professional education on LGBT social and health issues.^27^

However, it is important to note that over the past few of years, the VA has made significant strides in the increasing LGBT cultural competency and training by establishing their LGBT Program in the Office of Patient Care Services.^28^ For example, this VA program has developed three specific webinars about transgender veteran care, providing information about general transgender care (e.g., preferred name, pronoun usage), transgender mental healthcare and treatment, and the prescription and monitoring of hormone therapy. These webinars are available to all VA providers and employees through an online VA-sponsored education system.^29^ Also, the VHA has piloted a national program providing a 7-month intensive training on transgender care to interdisciplinary teams of clinicians to increase provider competency and expand clinical capacity.^30,31^ These educational trainings and system-level interventions may be particularly valuable considering that training specific to transgender health has been found to improve medical providers' competency and shift their attitudes toward transgender patients.^31,32^ In addition, a VA team of health professionals recently developed a nationwide e-consultation program, in which an interdisciplinary team of VA providers can provide patient-specific consultation through patient medical records, as well as access an online repository of reference material about the transgender healthcare services that are available to all veterans.^31,33^ Finally, the VHA has established nine LGBT-specific clinical fellowships available across the United States to better mentor junior mental healthcare providers with specialized training and increased knowledge about LGBT client/patient care.^29^

Despite the considerable efforts taken to establish, expand, and improve healthcare for transgender veterans, to our knowledge, there have been no studies specifically exploring the patient-centered experiences of transgender veterans in accessing and utilizing transition-related, primary, and mental healthcare services in the VA healthcare system since the provision of transgender transition-related healthcare (2011). The aim of the current study is to address this gap in the literature by qualitatively exploring the experiences of transgender veterans using the VA healthcare. In addition, this study examines transgender veterans' knowledge of and attitudes toward the VHA coverage and provision of transition-related care. Ultimately, the findings of this study underscore the challenges and barriers transgender veterans face in accessing and utilizing VA care and suggest the continued need for patient-centered and healthcare system-level interventions to improve the uptake and implementation of transgender VHA healthcare.^2^

## Methods

### Participants and procedures

Transgender veterans were recruited using passive in-person recruitment at the 2013 Southern Comfort Conference (SCC) reception for transgender veterans. Interested participants were able to inquire about study participation and schedule an interview time directly with the research team members hosting an information table at the event (B.J.H. and J.T.B.). In addition, participants could sign up to be e-mailed more information after the event and to schedule a subsequent interview time. In-depth semistructured interviews lasting 45–60 min were conducted in-person by two interviewers (B.J.H. and J.T.B.) during at the September 2013 SCC in Atlanta, GA. Interviewers utilized narrative inquiry methodology following an experience-centered approach where the participants guided the interviewer through their experiences, rather than individual recollections or events.^34,35^ Using this approach, we inquired about participants' broader military enlistment motivations, military and transition history, as well as specific experiences accessing and using the VA healthcare system, knowledge about current transgender transition-related policies, and potential recommendations for policy changes based on patient experience (Table 1). Subsequent probing questions were specific to individual responses and invoked more in-depth detail or self-analysis of overarching inquiry questions. All interviews were digitally recorded and transcribed verbatim for thematic coding and analysis. All participants provided verbal informed consent before participation and were offered $20 remuneration for their participation. Verbal informed consent was used to allow participants to complete anonymous participation and no identifying information was collected throughout the study procedures. Also, participants were asked to use a pseudonym or preferred “nickname” that would be used during the interview. Participants were also asked to provide basic sociodemographic information regarding their transgender self-identity (i.e., transgender woman or transgender man), branch of service, and approximate years serviced. Pseudonyms and nicknames were removed from the data presented in this article. All study protocols and procedures were approved by the university's Institutional Review Board.

**Table 1.** **Initial Interview Questions**

Interview inquiry questions
1. What type of medical care or treatment have you sought at the VA in the past 2 years?
2. Can you describe your experiences in accessing healthcare at the VA?
3. Can you describe why you chose (to use or not use) the VA healthcare system for your healthcare?
4. What do you know about the Veterans Health Administration's current policies and practices regarding transgender veteran transition-related care?
5. What recommendations, if any, do you have for how the VA could improve care for transgender veterans?

VA, Veterans Affairs.

### Data analysis

Data for analysis included the transcripts of the digitally recorded in-depth interviews. The major themes presented in this article were identified using a thematic analysis approach.^36^ Two research assistants (K.R. and C.L.) independently coded all 11 final transcripts using ATLAS.ti© (version 7.5.7). Coding proceeded in two steps. First, the coders read through each transcript several times and used open coding to note initial codes by highlighting relevant data to develop an initial codebook.^36^ Next, the two coders met to evaluate their preliminary codebooks and initial themes.^36^ Any differences in codes were discussed until a resolution was met and a final codebook was produced, combining or collapsing overlapping themes. Finally, the two coders applied the agreed upon codes to represent themes in the full set of transcripts and inter-rater reliability statistics was calculated. During final coding, no new major themes were identified indicating that saturation was attained.^36^ This coding framework achieved inter-rater reliability with a Kappa score of 0.63. All quotations supporting the major themes presented in this article were selected in collaboration with the two coders and research team.

## Results

### Participant characteristics

A total of 11 transgender veterans completed in-depth interviews. Of these veterans, nine identified as transgender women (transwomen) and two identified as transgender men (transmen). Participants represented a range of military branches: five in the Navy, two in the Army, two in the Marine Corps, one in the Air Force, and one in the Coast Guard. The length of service varied among participants with an approximate range of 3–21 years. All participants reported accessing healthcare at the VA while identifying as transgender, with the majority of participants (*n*=9) specifically reporting accessing services after the 2011 VHA directive. Additional sociodemographic characteristics (e.g., race/ethnicity and age) were intentionally omitted to allow for anonymous participation.

### Core themes

Five inter-related themes were identified as key factors impacting the accessibility and quality of care transgender veterans received through the VA. These five themes include the following: (1) long delays in receiving care; (2) needing to travel to receive care; (3) lack of patient knowledge regarding the coverage of transition-related care; (4) insensitivity, harassment, and violence from providers; and (5) a general lack of knowledge about transgender patients and care among providers. These themes represent barriers to VA provision of general healthcare and transgender transition-related care among transgender veterans.

#### Long delays in receiving care

Across interviews, participants expressed experiencing long delays in receiving transgender transition-related care through the VA healthcare system. Some participants (*n*=4) mentioned that the providers they encountered at the VA had to refer them to more knowledgeable medical professionals—a process that took patients few weeks to several months to receive an appointment. One participant recounted the following:

*I said, I was seriously considering going full-time … I needed a referral, so my doctor at the VA put in a request. It took a few months to find someone there who was willing to work with me.* (Transwoman, Air Force '70–'91)

Another participant expressed that mental health professionals at the VA she encountered were overly hesitant to provide letters authorizing hormone therapy, which forced her and other patients she knew to wait a long time before beginning their hormonal transition:

*I'm talking to people who are [transgender] veterans, and they say that when they go [to the VA], they are sent to a counselor. They go to the counselor dressed [as their gender], and the counselor is not experienced enough, so they still haven't been prescribed hormones. I'm like “What?” I know a lot of transgender people, who, as soon as they walk in to see a counselor outside of the VA, the counselor goes “Oh, you've been living like that, going out in public dressed? There's your hormones. Come see me in six months.” A lot of them are not doing that in the VA.* (Transwoman, Navy '97–'07)

The interviewees suggested that these long delays not only present challenges to veterans who are actively using the VA transition-related care but also serve as a deterrent for transgender veterans who were considering using the VA healthcare system to transition. For example, the same participant expressed that she specifically delayed talking to her primary care physician about beginning hormone therapy because she did not have the time to commit to the long process others had experienced. Similarly, another participant stated that she had opted to use private providers rather than the VA for transition-related care because of the long delays:

*When I go into a VA setting … I see the long lines, I see the cattle calls waiting at the pharmacy, I hear how long it takes for appointments, and things like that … I walk into the VA and quite honestly, I'm like “There is no way.”* (Transwoman, Navy '92–'95)

#### Needing to travel to receive care

Participants expressed having to travel long distances to access transition-related care. One woman from Montana explained:

*Unfortunately, there is no endocrinologist in [hometown], so I had to go to Billings, Montana, which is [over 200] miles away to see an endocrinologist. The VA wouldn't pay for it … they, of course, would pay for my healthcare but they wouldn't pay for my travel.* (Transwoman, Navy '90–'93)

In another case, a participant who lived on the East Coast was told by her primary-care physician to look into traveling to the West Coast to receive transgender transition-related care through the VA healthcare system:

*[I am looking into] having a double orchiectomy. Basically, I'm thinking at this point, I would not be able to get it on the East Coast. My doctor mentioned that I might be better off talking to a doctor on the West Coast who deals with transgender issues.* (Transwoman, Navy '77–'88)

Extensive travel for transgender transition-related care presents a barrier for transgender veterans using the VA healthcare system, which can be both costly and time-consuming.

#### Lack of patient knowledge regarding the coverage of transition-related care

Another theme expressed by the participants was the lack of knowledge among patients about the VHA coverage of transgender transition-related care. At the time of the interviews—2 years after the 2011 VHA directive began providing transgender transition-related care—two participants were still unaware of the types of transgender healthcare services provided by the VA, in particular, the availability and coverage of hormone therapy and gender-related mental health services under their veteran insurance. Some participants attributed lack of patient knowledge to poor publicity or dissemination of coverage by the VHA and VA providers. For instance, one participant mentioned that she had only heard about the provision of transgender transition-related care through networks of transgender friends via social media rather than directly from her providers at the VA:

*I don't remember hearing about the directive at the time it came out. I heard about it later I think through my friends in this community of transgender veterans on Facebook.* (Transwoman, Navy '92–'95)

Two participants recommended that the VHA more widely publicize the availability of transgender transition-related care through the VA healthcare system, one stating the following:

*[The availability of transition-related care] is not publicized. I think some of that needs to have public recognition … there's a monthly or quarterly little newsletter that comes out. It should be big and bold on there that, “This is how we treat transgender people now. This is what we can do. This is what we can't do.”* (Transman, Marine Corps '90–'10)

#### Provider insensitivity, harassment, and violence

Insensitivity, harassment, and violence in VA settings were reported by participants. In particular, 7 of the 11 participants reported experiencing some form of insensitivity, harassment, or violence while accessing services at the VA. For instance, one woman recounted an experience with her primary care physician at the VA clinic, stating the following:

*I went to see my primary care physician [at the VA]. It got actually physically abusive … She goes, “How did you get these?” and just reached out and flipped my boob … I said, “If you ever touch me again inappropriately, there's going to be a huge problem here.”* (Transwoman, Marine Corps '76–'89)

This particular participant also noted that her primary care physician refused to close the blinds to ensure her privacy while she changed her clothes. Another participant described experiencing stigmatization in mental healthcare settings at the VA clinic as follows:

*Sitting down with those psychologists was a unique process. A lot of ancillary things come up. “Oh you know if you want to be passable, you need to lose weight” and those types of things … [there were also] initially some weird looks when I went in.* (Transwoman, Army '85–'05)

In addition, participants reported being misgendered in VA healthcare settings. One woman explained:

*I have received some services through the VA…[In the waiting room,] even though I was dressed according to my gender, they would announce [my legal name].* (Transwoman, Marine Corps '76–'89)

Other participants articulated that they were unable to get preferred names and gender pronouns included in medical records, which also contributed to misgendering.

*There's been some challenges [at the VA] as far as getting the right gender pronouns done, getting paperwork changed, getting the name change done, and that type of thing. It's been more of an administrative hassle than anything else.* (Transwoman, Army '85–'05)

Also, this participant recommended that the VA creates fields in the medical record systems for patients' preferred names and gender pronouns.

#### Lack of provider knowledge

Nearly all participants (*n*=10) reported that VA healthcare providers were, for the most part, unknowledgeable about transgender transition-related care.

*The VA is … trying very hard, but they don't have the expertise yet. It's a brand new thing to them. So I'm seeing another psychiatrist that is learning as much as I am.* (Transwoman, Airforce '70–'91)

Participants also noted that they frequently had to educate their medical and mental health providers about transition-related care.

*My primary care physician at the VA has no clue. I've done more educating of her than anything else. You run into a lot of that with the doctors in the VA—that you're doing more educating with them. They have no clue about the hormones, why you were being prescribed the different hormones, and what effects they have and that type of thing.* (Transwoman, Army '85–'05).

Several participants (*n*=9) recommended that medical and mental health professionals, as well as VA frontline and support staff, undergo cultural competency training. One woman emphasized the need for explicitly comprehensive training.

*There needs to be a comprehensive training program. We need to take the time and spend the money to educate staff … I went to 20 different endocrinologists, and they were all like, “Hey, we don't understand this any better than you do.”* (Transwoman, Army '85–'05)

Another participant articulated that the VA should better connect providers with educational and informational resources about transition-related care provision.

*There's very few qualified people across the country who have the expertise to handle [transition-related care]. And so we need to do more to educate [VA] professionals … on how to deal with this … [and familiarize them] with the resources that are out there…I just don't know that they know where to go for resources or information.* (Transwoman, Army '92–'95)

## Discussion

Collectively, these experiences of transgender veterans emphasize the importance of formalized evaluation and training within the VA healthcare system to address the needs of transgender veterans, particularly as the VA represents one of the largest healthcare provider networks of transgender people.^3,5,15–23^ To our knowledge, our qualitative study is one of the first to specifically explore the patient-centered experiences of transgender veterans accessing and utilizing VA healthcare and transgender transition-related care since the VHA directive explicitly began providing transgender healthcare services in 2011. Overall, our findings suggest that transgender veterans continue to face challenges and barriers in accessing culturally competent and informed transgender transition-related care at the VA. These challenges include long delays in receiving care, the need to travel to receive care, lack of knowledge about transgender transition-related care coverage by the VHA, insensitivity, harassment, and violence in VA clinics, and a general lack of knowledge about transgender patients and care among providers. To address some of these challenges, transgender veteran participants highlighted several key areas for intervention to improve healthcare provision and subsequent patient-centered health outcomes. In particular, our participants suggested that the VHA more widely publicize the availability of transgender transition-related care, so that transgender veterans, especially those who utilize the VA for primary healthcare coverage, are aware of the VA care. The VHA has recently taken steps in this area by clearly outlining the availability of transition-related care on VA websites and developing public campaigns regarding LGBT inclusion, including the “We Serve All Who Served” campaign.^37–39^ Research examining awareness of the VA policies and services regarding transgender care among transgender veterans may be useful in assessing the potential impact and reach of such efforts. Participants' healthcare system-level recommendations focused on creating entry fields for preferred names and gender pronouns in electronic and paper medical records, increasing training for staff and medical and mental health providers, and better connecting VA providers with information and resources concerning transition-related care. Of these recommendations, the VHA has already begun to develop and implement trainings as well as resources for VA providers to access information and consultation regarding transgender care.^29–31,33^ Future studies evaluating the system-level efficacy of these interventions with a particular emphasis on patient experience would be valuable to better understand the success of their implementation.

Given that the VA has a “zero-tolerance standard for harassment of any kind,” the prevalence of insensitivity, harassment, and violence articulated by participants in this study suggest that the VA may need to re-examine its current monitoring and reporting procedures for such adverse events.^1,2^ In addition, VA clinics may need to take additional steps to increase the availability of the transgender transition-related care they provide in an effort to address long delays and mitigate the need for transgender veterans to travel extensively. Currently, the VA has a non-VA Medical Care Program in place, which authorizes veterans to seek care from non-VA providers when wait times for VA appointments or the travel required to access VA facilities is impractical.^40^ However, our data suggest that the VA process for referring patients to outside providers may not adequately address issues regarding delays or necessitated travel as some participants experienced long wait times during the referral process itself or were referred to distant providers to received transgender transition-related care, underscoring the need for quality management assessment specific to LGBT healthcare in the non-VA Medical Care Program.^40^

Overall, the findings of our study converge on the importance of improved awareness, access, and quality of VA healthcare for transgender veterans through system-level intervention. Furthermore, our findings underscore the need for additional research assessing patient awareness of VHA policies and VA services, the quality of transgender transition-related care, incidence of adverse events (e.g., insensitivity, harassment, and violence) at the VA in a larger sample of transgender veterans, experiences and perspectives of VA medical and mental healthcare providers who serve transgender veterans, and impact of recent VA interventions such as training and consultation programs on transgender patient experiences. Improving the access and quality of care for transgender veterans utilizing VHA benefits is critical given that transgender people are overrepresented in the veteran community and that the VA may be the single largest transgender serving healthcare system in the United States.^3,5,15–23^ In addition, since research suggests that transition-related care alleviates distress associated with gender identity and body incongruence for many transgender people, the effective uptake and implementation of VA transition-related care may be especially important in addressing some of the stark mental health disparities experienced by transgender veterans.^11–14^

Moving forward, as the VA develops and expands programs for transgender patients, continued patient-centered evaluation will be critical. Also, examining the implementation of these programs across the United States will be important to ensure that access to gender-affirming care for transgender veterans in varying regions is equitable. Finally, as the Department of Defense continues to evaluate the current ban on open transgender service, future adjustments to existing VA programs and interventions may needed to meet the needs of transgender veterans who may have transitioned before, or during their military service, which would require additional education on lifelong transgender healthcare and transgender aging.^41^

### Limitations

Although this study provides insight into the patient experiences of transgender veterans in the VA system, it is not without limitations. Notably, this qualitative study is limited in that it relies on a relatively small convenience sample of transgender veterans. Although thematic saturation was achieved during in-depth interviews, the interviews do not necessarily reflect the views and experiences of all branches of service and all transgender veterans. Additional factors, such as number of deployments, military rank, and length of service, may contribute to various degrees of knowledge about VHA directive changes or interest in VA transgender transition-related care. We did not collect information regarding the location of VA healthcare clinics accessed by our participants, although those mentioned by participants differed by city and state. Thus, this study cannot draw direct conclusions or make recommendations for any specific VA clinic or VA medical protocol, and study findings may not be generalizable to, or representative of, all VA healthcare clinics in the United States. Although most participants reported accessing services through the VA within the past 2 years, we did not collect data regarding the length since last VA visit or visit frequency. In addition, given that participants were recruited at the SCC (Atlanta, GA), participants may be more likely to publically identify as transgender and have connections to transgender community, than samples of transgender veterans recruited through VA clinics. Another limitation is that participants were accessing transgender transition-related care through the VA at different points in their medical transition and had different long-term transition goals (e.g., hormone therapy only vs. gender affirmation surgery). Additional studies addressing these limitations are needed to fully examine the larger ecology of transgender veteran healthcare service.

## Implications

On June 9, 2011, the VHA issued its first directive to provide transgender and intersex veterans with healthcare services through the VA, particularly hormone therapy and gender-related mental health counseling.^1,2^ In addition, the VHA mandated that the VA healthcare system provide care without discrimination and in support of veterans self-identified gender.^1,2^ To date, few studies have explored the experiences of transgender veterans who have accessed transgender transition-related healthcare services through the VA healthcare system. Thus, this study offers a valuable perspective on the patient experience of transgender veterans in accessing and utilizing care at VA clinics. Overall, the study findings suggest that barriers and challenges to accessing transgender transition-related healthcare services through the VA healthcare system persist. Furthermore, study findings and transgender veteran patient recommendations urge that medical and cultural competency training among VA medical and mental health professionals may be critical to successfully implementing VA transgender care and ultimately improving the health of the US transgender veteran population. Ultimately, as policies, programs, and patient needs shift, continued patient-centered evaluation and necessary adjustments may be critical to ensuring accessible, high-quality care for transgender veterans.

## Abbreviations Used

GID: gender identity disorder
SGM: sexual and gender minority
TAVA: Transgender American Veterans Association
VA: Veterans Affairs
VHA: Veterans Health Administration
